# Preliminary experience with the micro vascular plug for the treatment of pulmonary arteriovenous malformation: case series of four patients

**DOI:** 10.1186/s42155-018-0027-z

**Published:** 2018-09-28

**Authors:** Stevo Duvnjak, Carmela Anna Di Ciesco, Poul Erik Andersen

**Affiliations:** 1 Department of Radiology, Odense University Hospital, Sdr. Boulevard 29, DK-5000 Odense C, Denmark; 2 Department of Clinical Research, University of Southern Denmark, Odense, Denmark

**Keywords:** MVP, Microplug, PAVM, Embolisation

## Abstract

**Background:**

To describe the preliminary experience using Micro Vascular Plug (MVP) for treatment of pulmonary arteriovenous malformations (PAVMs) with small feeding arteries (3 -5 mm) in four patients with Hereditary Haemorrhagic Telangiectasia (HHT).

**Material and methods:**

One female and three male patients with PAVMs have been treated during 2017. The mean age was 32.5 years; (range: 20–53). All patients underwent contrast echocardiography and computed tomography of the chest to establish the diagnosis.

**Results:**

Four patients with PAVMs were treated with embolisation using the MVP-3Q and MVP-5Q micro plugs. All MVP were placed without complications and with following immediate occlusion of the PAVMs in all three cases. In one case MVP was placed semi selectively. All cases were with the good clinical outcomes.

**Conclusion:**

MVP is a new detachable embolisation material which is easy to use. Maximal control during the deployment and immediate occlusion of the target vessels can be achieved.

## Background

Hereditary Haemorrhagic Telangiectasia (HHT) is an autosomal dominant disease, frequently associated with arteriovenous malformations in the brain, lungs, gastrointestinal tract and liver (Faughnan et al., 2011). Pulmonary arteriovenous malformations (PAVMs) are anomalous communications between pulmonary arteries and veins and occur in 15–50% of people with HHT (Faughnan et al., 2011; Kjeldsen et al., 2000). They have one or more feeding arteries, an aneurismal sac and one or more draining veins; they can be simple (with one feeding artery) or complex (with more than one feeding artery).

PAVM can be associated with life-threatening complications, like transient ischemic attack (TIA), stroke, cerebral access, haemoptysis and haemothorax. Neurological complications occur via paradoxical embolisation (Cottin et al., 2004; Cartin-Ceba et al., 2013), while haemorrhagic pulmonary complications can occur due to rupture of PAVMs which is very rare, described in only a few cases (Kundu et al., 2010).

Screening for PAVMs has to be performed at the time of the initial evaluation for HHT using contrast echocardiography (CE) (Parra et al., 2010). CE is considered positive if there is detection of any bubbles in the left atrium; positive CE has to be followed by computed tomography (CT) of the thorax.

PAVMs are usually treated with embolisation to prevent complications (Iqbal et al., 2000, Moussouttas et al., 2000). Treatment aims to occlude the feeding artery as close as possible to the sac. Several devices have been used during the years (balloons, coils, microcoils, vascular plugs) with the generally good clinical outcome. However, recanalisation of embolized PAVM can occur in between 5 and 15% (Milic et al., 2005). Microvascular plug (MVP) is a new tool in the embolisation toolbox. It is a detachable nitinol skeleton plug, coated with polytetrafluoroethylene (PTFE) (Pellerin et al., 2014). There are potential advantages of using MVP, such as the ability to deploy through a microcatheter close to the PAVM sac with immediate occlusion. Further, the device can be retracted and replaced, less metal artefact compared to coils, possibility to prevent device migration and less probability to have recanalisation because of the PTFE coating (Boatta et al., 2017).

There are two sizes of MPVs: MPV-3Q used for arteries ≤3 mm or less in size, and MPV-5Q for arteries of 3–5 mm. Both microplugs are designed to be delivered through microcatheters, MPV-3Q is compatible with microcatheter of 2.4 Fr (0.021-in. inner lumen diameter), and MVP-5Q is compatible with 2.8 Fr (0.027-in. inner lumen diameter).

This preliminary report aims to describe the intraprocedural experience using MVP in the treatment of PAVM.

## Material and methods

Three female and one male (mean age 32,5 years; range: 20–53) with PAVMs diagnosed on CE and CT have been embolized with MVPs.

### Embolization procedure

Odense University hospital is the Danish national centre for diagnosis and treatment of HHT patients. Patients with PAVMs are generally treated with embolization if they have PAVM with ≥3 mm feeding artery, and in some cases even smaller (Andersen and Kjeldsen, 2012).

The embolizations have been performed in local anaesthesia via the right femoral vein through a 7-F sheath. All PAVM embolizations have been performed in a bi-plane angio-room (Artis zee biplane, Siemens AG Erlangen,Germany). 70 UI/per kg of heparin was administrated in a bolus. No antibiotics were given during the embolization. 7 Fr 100 cm long ConsierGE guiding catheter (MeritMedical, South Jordan, UT, USA) and coaxially advanced 5 Fr 125 cm long Bernstein shape catheter (MeritMedical, South Jordan, UT, USA) was navigated to the pulmonary artery. The angiography confirmed the PAVM and automatic vessels measurement using the software (Syngo QVA vascular analysis) of the feeding artery was performed. Microcatheter either 2.4 Fr or 2.8 Fr was advanced coaxially into the feeding artery as close as possible to the PAVM and MVP was deployed. The patients were admitted to the hospital and discharged within 24 h after the intervention.

### Follow-up

Clinical and CE follow-up was performed in three cases and only clinical examination in one case. The mean follow-up period was 15.5 months.

### Ethics

All procedures in the studies involving human participants were performed according to the ethical standards of the institutional and national committee. The local data protection agency approval with the journal number 17/14191.

## Cases presentation

### Case 1

**Fig. 1:**
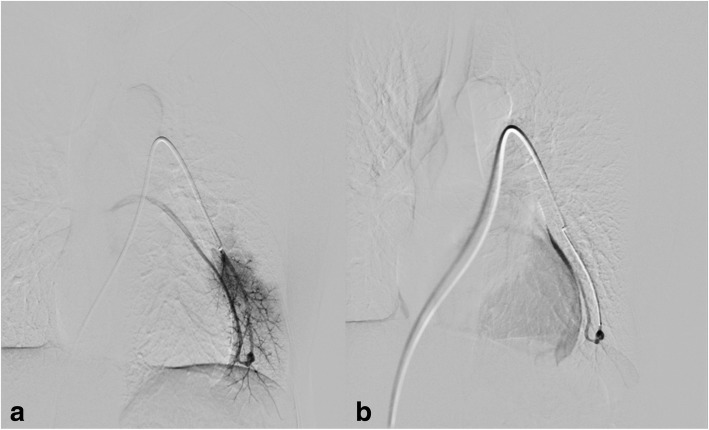
**a** Baseline pulmonary digital subtraction angiography in a 52-year-old woman, affected by HHT, with several small PAVMs. **b** The biggest of these was located in the left lower lobe, with a feeding artery of 3 mm

**Fig. 2:**
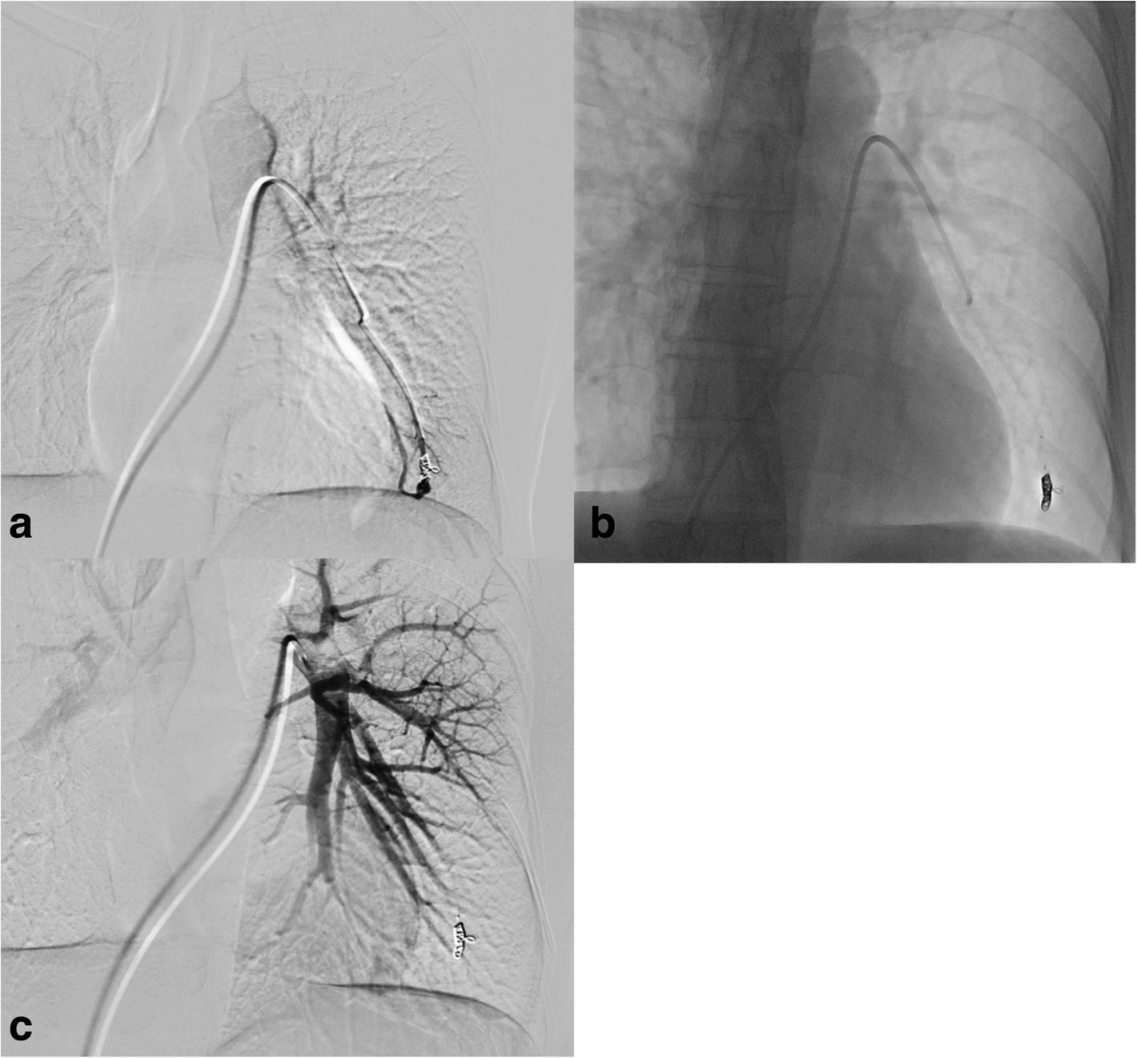
**a** After coil deployment, there was still flow in PAVM. **b** and **c** An additional MVP-3Q was deployed, and the flow stopped immediately

A-52-year-old female with HHT. Saturation at rest was 96% and during exercise decreased to 92%. CE confirmed a shunt with grade I-II. CT without contrast confirmed a simple PAVM in the left lower lobe with a feeding artery of 3 mm. The left pulmonary artery was catheterised, and angiography depicted the PAVM (Fig. 1a, b). The PAVM was first embolised with a detachable coil of 4 mm diameter and 10 cm in length (Interlock, Boston Scientific Marlborough, MA, USA). After ten minutes waiting there was still flow thought the PAVM. It was decided to deploy an MVP-3Q (Reverse Medical Corporation, Irvine CA, USA) through a 2.4 Fr microcatheter (Renegade microcatheter, Boston ScientificMarlborough, MA, USA) with following immediate occlusion (Fig. 2a-c). No complications occurred during the intervention. The patient was discharged the following day, and follow-up consists of clinical and CE control which showed improved oxygenation and no shunt at CE control six months after embolization.

### Case 2

**Fig. 3:**
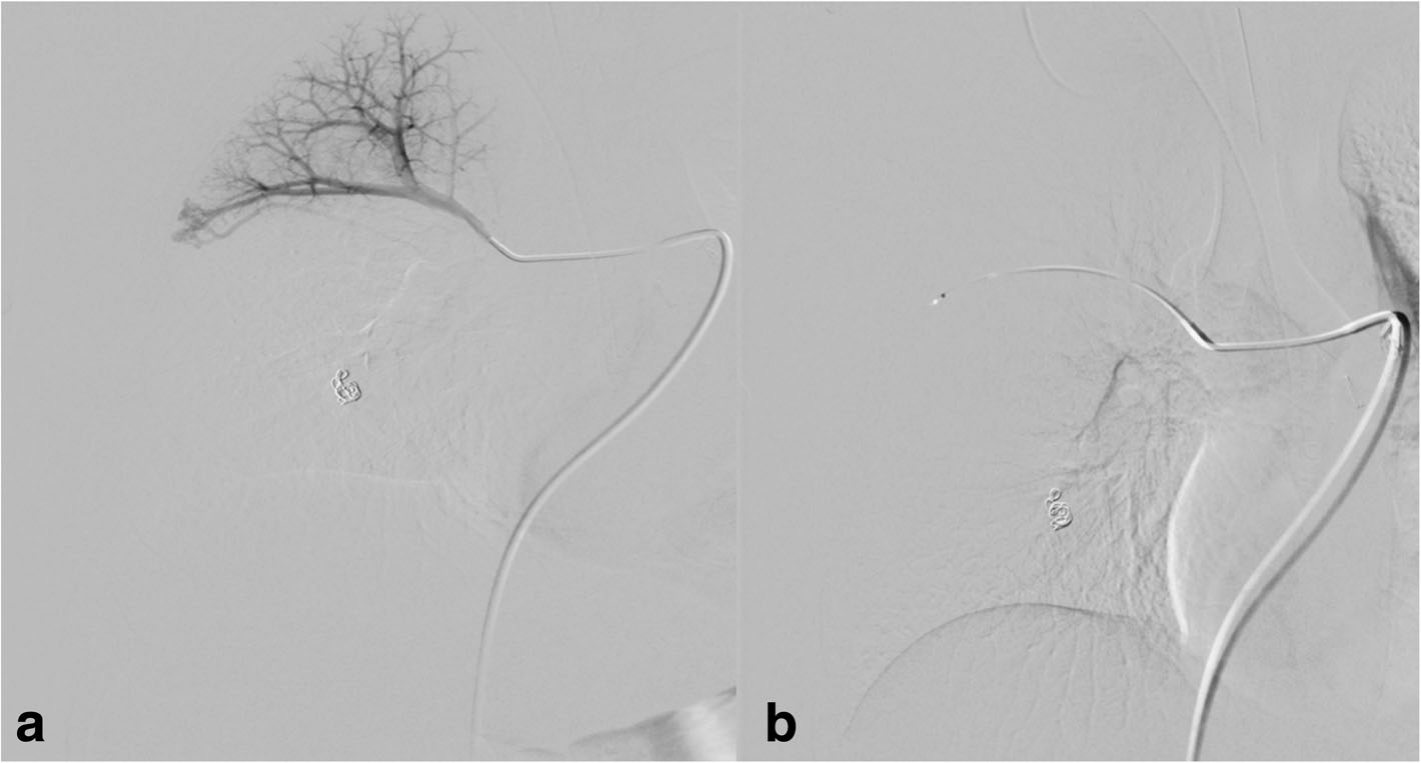
**a** A 19-year-old woman with HHT. Small PAVM with feeding artery 3.2 mm in the right upper lobe. **b** Previous embolization with coils of complex PAVM in the same lobe

**Fig. 4:**
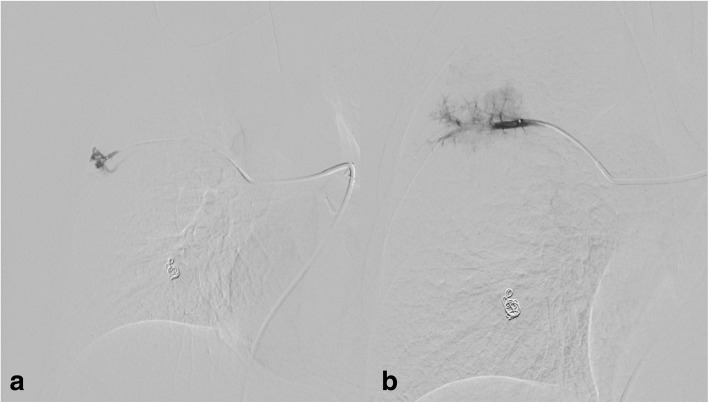
**a** Before deployment of MVP-5Q. **b** After deployment of MVP-5Q, immediate stop of flow

A-19-year-old female with HHT and haemoptysis underwent three PAVM embolizations previously. The last embolization was performed in 2009, and the patient was without complaints. Recently she complained about shortness of breath and pain during mild exercise. Saturation at rest was 92% and during the exercise decreased to 89%. CE confirmed a shunt grade I-II. CT without contrast confirmed two new PAVMs. The right pulmonary artery was catheterized, and angiography depicted two PAVMs, one in a right upper lobe and one in the right lower lobe (Fig. 3a, b). Both PAVMs had feeding arteries with a diameter of 3.2 mm. The PAVM in the lower lobe was embolized with a detachable coil (Interlock, Boston ScientificMarlborough, MA, USA), 6 mm in diameter and 10 cm long. The PAVM located in the upper lobe was engaged coaxially with microcatheter 2.8 Fr (Renegade Hi-Flo; Boston ScientificMarlborough, MA, USA) and primarily embolized with MVP-5Q (Reverse Medical Corporation, Irvine CA, USA) with following immediate occlusion (Fig. 4 a, b). No complications occurred during the intervention. The patient was discharged the next day, and follow-up consisted of clinical and CE control showed improved oxygenation up to 96% and no shunt at six months CE control.

### Case 3

**Fig. 5:**
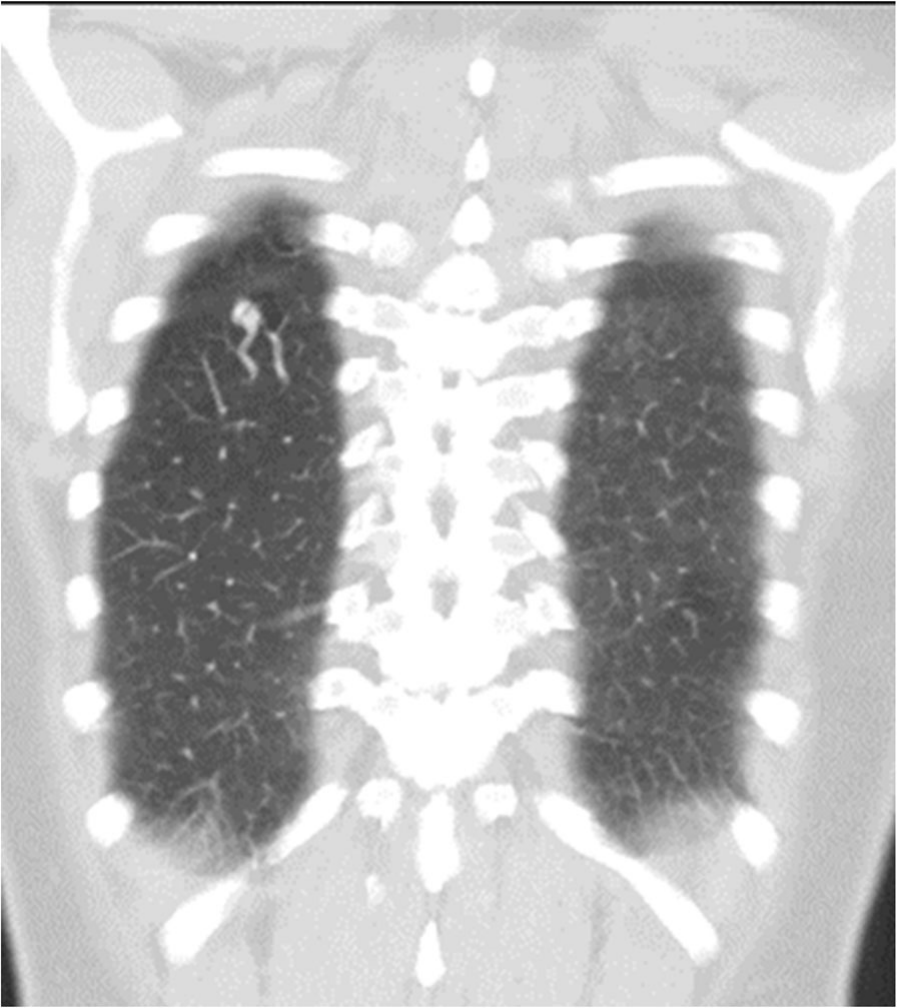
Non-contrast CT showed a simple PAVM in right upper lobe with feeding artery of 3, 3 mm in diameter

**Fig. 6:**
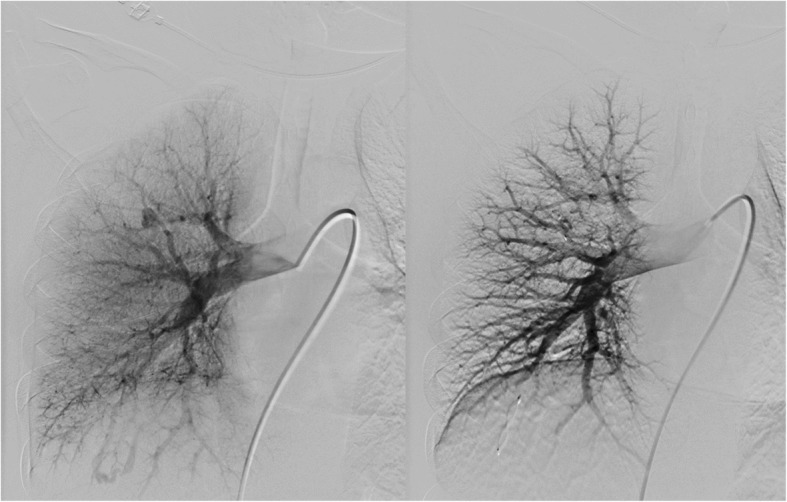
Pulmonary angiography before and after embolization of both PAVMs in the right lung. The PAVM in upper lobe was embolized with MVP-5Q

A-32-year-old female with nasal bleeding during two years. A diagnosis of HHT was established. Oxygen saturation was 98% without significant changes during exercise. CE confirmed shunt grade II-III and non-contrast CT showed two PAVMs, one located in the right lower lobe with feeding artery of 5 mm and the other with feeding artery of 3.3 mm in diameter located in the right upper lobe (Fig. 5). The PAVM in the lower lobe was embolized with Amplatzer plug IV (St Jude Medical, Minnesota, USA), 8 mm in diameter. The PAVM in the upper lobe was embolized with MPV-5Q (Reverse Medical Corporation, Irvine CA, USA) delivered through a microcatheter 2.8 Fr (Renegade Hi-Flo; Boston ScientificMarlborough, MA, USA) with following immediate occlusion of both PAVMs (Fig. 6 a, b). No complications occurred during the deployment. The patient was discharged the next day, and clinical control and CE showed no shunt six months after embolization.

### Case 4

**Fig. 7:**
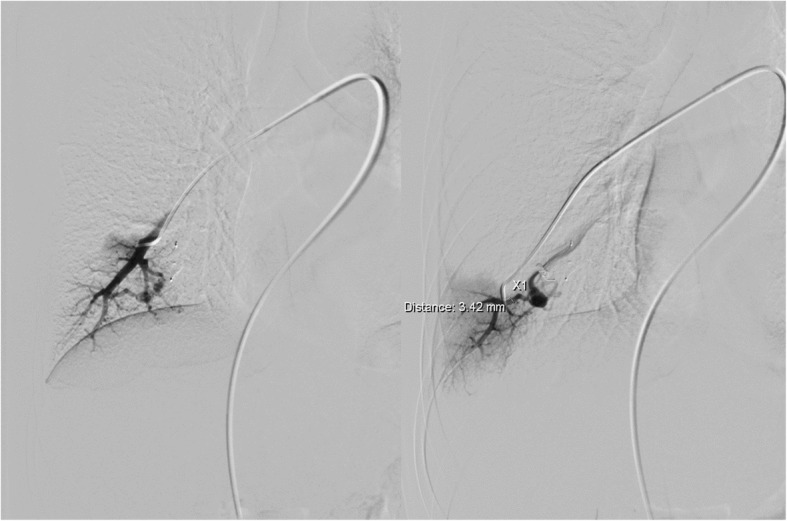
Pulmonary angiography before embolization of PAVMs in the right lung with two feeding arteries

**Fig. 8:**
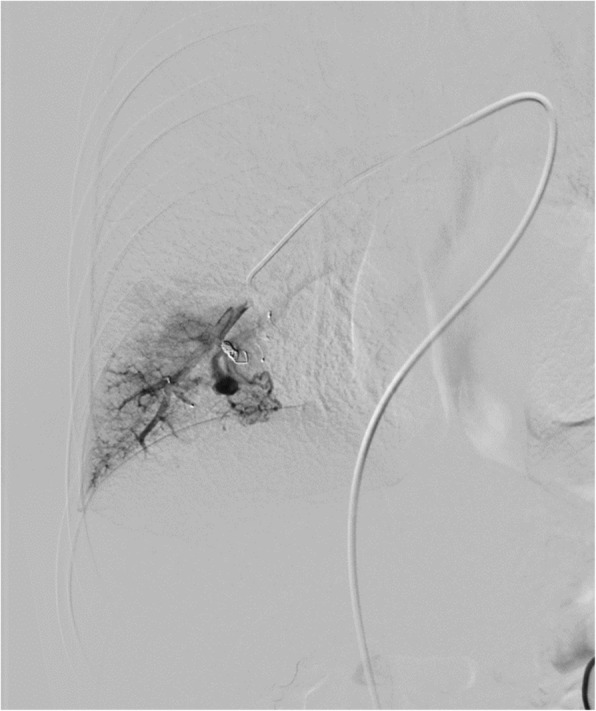
The PAVM in lower lobe was embolized with detachable coil and with MVP-5Q

20-year-old male patient with diagnosed HHT and multiple PAVMs in both lungs. Embolization of PAVMs in the left lung was performed in 2010 and 2011 with a good outcome. In 2017 CE control showed shunt grade II-III and CT confirmed two PAVMs in the right lung. The biggest had a feeding artery of 6 mm and was embolized with Amplatz plug IV (St Jude Medical, Minnesota, USA), 8 mm in diameter a few months before the actual intervention (Fig. 7). The smaller PAVM in the right lower lobe had two feeding arteries. One feeding artery with a diameter of 3 mm was embolized with a detachable coil 4 mm and 8 cm in length (Interlock, Boston ScientificMarlborough, MA, USA). The other feeding artery with a diameter of 3.4 mm was embolized with MVP-5Q delivered through a microcatheter 2.8 Fr (Renegade Hi-Flo; Boston ScientificMarlborough, MA, USA), but due to highly angulated feeding artery the MVP was displaced a little proximally and occluded the feeding artery a longer distance to the PAVM than intended (Fig. 8). No complications occurred, and the patient was discharged the following day without symptoms. The patient was asymptomatic at clinical control 12 months after the embolization, and no CE was performed during the follow-up. The patient did not experience any symptoms during the 12 months follow-up period.

## Discussion

This case series described an early experience with the new embolization material and showed high technical success and control during the embolization of four PAVMs. The main advantage of MVP is a possibility to deploy the plug through the microcatheter, and if the planning is accurate, only one MVP is needed to achieve complete occlusion. One important feature of the new device is that we need straight at least 12 mm long vessel to deploy the MVP precisely. In the case of vessel tortuosity and sharp take off of the branch vessels, the deployment can be more challenging. The best results are achieved when the embolization material is placed close as possible to PAVM sac and where the distance between the PAVM sac and embolization material is under 1 cm, especially in PAVM with small feeding artery as reported by (Stein et al., 2017). They found the higher rate of reperfusion of the PAVM in a case of the proximally placed embolization material. The present case with difficulties to deploy the microplug close to the PAVM sac in tortuous anatomy showed the potential limitation of this new device. The possible combinations of the microcoils and microplug can be a solution in such environment. However, the further study can confirm the value of such an approach.

The present reports in the literature have the same experience with the MVP (Boatta et al., 2017; Pellerin O et al., 2014; Conrad et al., 2015).

The possible migration of the device is mentioned as a complication because of the undersized diameter (Conrad et al., 2015). We did not have any distal migration as we followed instruction for use, where the vessels with a diameter of ≤3 mm should be embolized with MVP-Q3 and vessels with diameter 3–5 mm should be embolized with the MVP-5Q. However, in the present cases report CT control was not performed to evaluate device migration eventually. Other authors recommend coils placement additionally in a case of incomplete occlusion or in a case of plug migration (Trerotola S et al., 2010). The potential challenge with this technique using the MVP-5Q is that the microcatheter has to be exchanged to the 0.021-in. inner lumen to avoid jamming of the coils when pushing coils thought a 0,027-in. inner lumen microcatheter. It is very important to avoid or to reduce manipulation and exchange of the material to avoid thrombus formation eventually, and air embolism and pre-interventional planning are very important. The CT and angiography measurement before advancing the embolization material is usually sufficient to choose the appropriate material and avoid too much manipulation.

## Conclusion

MVP embolization of PAVMs appears technically feasible, safe, and effective at early follow-up.

Further prospective studies on bigger populations are required to confirm long-term safety and efficacy of this promising new embolization material.

## Abbreviations

CE: Contrast echocardiography
CT: Computed tomography
Fr: French
HHT: Hereditary Haemorrhagic Telangiectasia
MVP: Micro Vascular Plug
PAVM: Pulmonary arteriovenous malformations
PTFE: Polytetrafluoroethylene
TIA: Transient ischemic attack
