# Correction to: Preliminary experience with the micro vascular plug for the treatment of pulmonary arteriovenous malformation: case series of four patients

**DOI:** 10.1186/s42155-019-0092-y

**Published:** 2020-01-08

**Authors:** Stevo Duvnjak, Carmela Anna Di Ciesco, Poul Erik Andersen

**Affiliations:** 1 Department of Radiology, Odense University Hospital, Sdr. Boulevard 29, DK-5000 Odense C, Denmark; 2 Department of Clinical Research, University of Southern Denmark, Odense, Denmark

**Correction to: CVIR Endovasc (2018) 1:19**

**https://doi.org/10.1186/s42155-018-0027-z**

In the published article (Duvnjak et al. 2018) the statement under the subheading ‘Consent for publication’ is incorrect.

“Not applicable”

should read:

“Informed consent for publication of this case report and its accompanying images has been obtained from the patient”

## References

[CR1] Duvnjak S, Di Ciesco CA, Andersen PE (2018) Preliminary experience with the micro vascular plug for the treatment of pulmonary arteriovenous. CVIR Endovasc 1:19. 10.1186/s42155-018-0027-z 10.1186/s42155-018-0027-z PMC6319526 30652150

